# Longitudinal Changes in Resting Metabolic Rates with Aging Are Accelerated by Diseases

**DOI:** 10.3390/nu12103061

**Published:** 2020-10-07

**Authors:** Marta Zampino, Majd AlGhatrif, Pei-Lun Kuo, Eleanor Marie Simonsick, Luigi Ferrucci

**Affiliations:** National Institute on Aging, National Institutes of Health, Baltimore, MD 21224, USA; marta.zampino@nih.gov (M.Z.); majd.alghatrif@nih.gov (M.A.); perry.kuo@nih.gov (P.-L.K.); simonsickel@grc.nia.nih.gov (E.M.S.)

**Keywords:** resting metabolic rate, chronic diseases, aging, body composition

## Abstract

Resting metabolic rate (RMR) declines with aging and is related to changes in health status, but how specific health impairments impact basal metabolism over time has been largely unexplored. We analyzed the association of RMR with 15 common age-related chronic diseases for up to 13 years of follow-up in a population of 997 participants to the Baltimore Longitudinal Study of Aging. At each visit, participants underwent measurements of RMR by indirect calorimetry and body composition by DEXA. Linear regression models and linear mixed effect models were used to test cross-sectional and longitudinal associations of RMR and changes in disease status. Cancer and diabetes were associated with higher RMR at baseline. Independent of covariates, prevalent COPD and cancer, as well as incident diabetes, heart failure, and CKD were associated with a steeper decline in RMR over time. Chronic diseases seem to have a two-phase association with RMR. Initially, RMR may increase because of the high cost of resiliency homeostatic mechanisms. However, as the reserve capacity becomes exhausted, a catabolic cascade becomes unavoidable, resulting in loss of total and metabolically active mass and consequent RMR decline.

## 1. Introduction

Resting metabolic rate (RMR) changes over the life span and has been related to changes in health status. RMR reflects the energy expended by the human body in a prolonged resting state in the absence of food digestion, physical or cognitive activities [1]. As such, RMR can be understood as the “cost of living”, i.e., the energetic cost of maintaining all physiological processes that preserve homeostatic equilibrium and cognitive alertness and sets the stage for all activities of life. RMR is affected by changes in body size, with greater RMR associated with larger body size, especially large lean body mass [2,3]. RMR is widely determined by the most metabolically active tissues, such as muscle, heart, brain, and liver, and, as the function and metabolic activity of these organs and tissues decline with aging, RMR also declines with aging [4]. The decline of RMR with aging has been partially, but not completely, explained by changes in body composition, and by the decline of lean mass more than by that of fat mass. This phenomenon is particularly evident in the years prior to death, when the drop of both lean and fat mass accelerates [5,6]. A lower RMR is observed in women, and this finding too has not been completely explained by differences in body composition. It has been suggested that the changes in body composition and parallel changes in RMR are due to the emergence of pathology, although the specific mechanism and directions of this association are still not understood.

In an analysis of data from the Baltimore Longitudinal Study of Aging (BLSA), subjects in good health and functional status had lower RMR than those affected by chronic diseases and functional limitations, independent of age, sex and body composition [7]. Also, independent of relevant confounders, higher RMR was cross-sectionally associated with both a higher number of chronic diseases and with significantly higher risk of developing multimorbidity, defined as two or more out of 15 chronic conditions [8]. Similarly, in community-dwelling women 60 years and older, increasing multimorbidity was associated with an increase in RMR independent of body composition and age [9]. Moreover, two studies evaluating the longitudinal association between energy metabolism and mortality found higher RMR and 24 h energy expenditure (24EE), which are predictive of future negative health outcomes and early mortality [10,11].

Overall, these data suggest that while healthy aging is associated with a progressive decline of RMR, independent of changes in body composition, superimposed adverse changes in health and functional status tend to attenuate such decline. This attenuation has been attributed to the potential extra-energetic cost of maintaining homeostasis in response to disease-related processes [12]. However, a comprehensive analysis of how various diseases that ensue with aging affect age-associated changes in RMR is still lacking. In this analysis, we aimed to assess whether having specific age-associated diseases, or the development thereof, correlate with RMR changes over time. Information on such associations is important to understand the energetic burden of specific diseases and whether early perturbation in energetic metabolism can inform impending health deterioration and future pathology.

## 2. Materials and Methods 

### 2.1. Participants 

Participants were community-dwelling persons recruited from the Baltimore and Washington DC areas. The BLSA has been continually recruiting healthy participants since 1958 and following them indefinitely regardless of any intervening change in health and functional status. The visit frequency depends on age: visits occur every 4 years until the subject reaches age 60, biannually from the age of 60 to 79, and yearly thereafter. A description of the sample and selection criteria has been previously reported [3,13].

The sample for the current analysis consists of 997 persons aged 24 to 95 years followed from March 2006 to December 2019. All participants underwent a comprehensive medical history interview, clinical examination, laboratory and cardiovascular testing, respiratory testing, cognitive testing, total body dual-energy x-ray absorptiometry (DEXA) imaging, and RMR assessment. Standard questionnaires inquired about different types of activities performed during a typical week, and the measure of physical activity considered encompassed all the high intensity activities, including brisk walking [14].

### 2.2. Chronic Diseases 

Fifteen conditions that occur with higher frequency and are associated with high disability and mortality risk in the aging population were selected “a priori” for this study and included: chronic heart failure (CHF), myocardial infarction (MI), cerebrovascular accidents (stroke or transient ischemic attack), hypertension, type 2 diabetes mellitus (T2D), anemia, peripheral artery disease (PAD), cognitive impairment, depression, Parkinson’s disease (PD), chronic kidney disease (CKD), cataract, chronic obstructive pulmonary disease (COPD), cancer, and osteoarthritis (OA). The presence of these conditions was assessed at baseline and every follow-up. 

CHF was defined as ejection fraction < 40%, assessed through echocardiography. Diagnosis of hypertension was based on measured systolic blood pressure ≥ 140 mmHg or diastolic blood pressure ≥ 90 mmHg, or self-report history of taking anti-hypertensive medications. T2D was defined as having: (1) history of T2D or hypoglycemic treatment; or (2) fasting plasma glucose (FPG) ≥ 126 mg/dl and 2-hr post oral glucose tolerance test (OGTT) plasma glucose (2hrG) ≥ 200 mg/dl at the same visit; or (3) FPG ≥ 126 mg/dl or 2hrG ≥ 200 mg/dl during two consecutive visits, in accordance with American Diabetes Association criteria. PAD was defined as ankle-brachial index (ABI) measured by Doppler stethoscope < 0.9 [15]. Cognitive impairment was assessed with the Mini-Mental Status Exam (MMSE), with impairment defined as score < 24 [16]. Depression was diagnosed with a Center for Epidemiologic Studies-Depression Scale (CES-D) score ≥ 16 [17]. CKD was defined using as the glomerular filtration rate an eGFR value < 60 mL/min estimated by the MDRD formula: 175 × (Creatinine clearance) ^−1.154^ × (Age) ^−0.203^ × (0.742 if female) × (1.210 if black) [18]. Anemia was defined as circulating levels of hemoglobin less than 13 g/dL in men and 12 g/dL in women. A past or current history of MI, CHF, cerebrovascular accidents, PD, COPD, cataract, cancer (with the exclusion of benign tumors and non-melanoma skin cancers), and OA was ascertained through a standardized questionnaire administered by trained personnel. 

### 2.3. Resting Metabolic Rate

RMR was estimated using indirect calorimetry (Cosmed K4b2, Rome, Italy) [19,20] evaluated first thing in the morning, shortly upon awakening, after an overnight stay in the clinic in a quiet, thermoneutral environment. Measurements lasted 16 min. Participants were in a fasting state and avoided ingestion of common stimulants, such as coffee and tea. Before testing, the analyzer was calibrated using a 3.0-L flow syringe and gases of known concentrations. The analyzer collects gas-exchange data on a breath-by-breath basis averaged over 30-s intervals to reduce variability. RMR in kilocalories per day was calculated from gas-exchange data using the Weir equation (1949) [21]. The first 5 min of data were discarded to allow adaptation to the testing procedures, and the remaining 11 min were averaged to obtain a single measure of RMR [22].

### 2.4. Body Composition 

Total body dual-energy x-ray absorptiometry (DEXA) was performed using the Prodigy Scanner (General Electric, Madison, WI) and analyzed with version 10.51.006 software. DEXA uses tissue absorption of x-ray beams to identify different body composition components (bone mineral content, lean body mass, and fat mass) and provide quantitative data on body composition [23,24].

### 2.5. Statistical Analyses 

Statistical analyses were performed using SAS version 9.4 and RStudio version 1.2.1335. Covariates considered in the models were sex (encoded as 1 = male, 0 = female), age, total body fat mass, and total body lean mass, since it is well known that these variables contribute to basal energy expenditure. Parameters such as smoking status, alcohol intake, physical activity level, height and weight, and subcutaneous or visceral distribution of the adipose tissue (assessed through computed tomography) were considered as possible covariates but then removed from the models when they did not contribute significantly to model fit. *p* < 0.05 was considered statistically significant.

Linear regression models were run to test the cross-sectional associations of RMR with the presence of each of the 15 selected conditions, adjusting for sex, age, lean body mass, and fat body mass as measured by DEXA.

Linear mixed effect (LME) models were used to estimate the longitudinal rate of change in RMR as described previously [25]. In summary, LME models were fitted for RMR using time since follow-up as the time scale with a random intercept and random slope. The rate of change (RMR_Change_), i.e., the individual specific slope, was calculated by adding the individual random effect coefficient (individual deviation from population slope) to the fixed effect coefficient (i.e., the population slope).

Multiple linear regression analysis was used to examine the determinants of RMR_Change_ with separate models fitted for each disease. Two hypotheses were evaluated: First, we tested whether participants affected by one specific chronic condition at baseline experienced different changes in RMR compared to those who did not have that condition at baseline (no disease). Secondly, we tested the hypothesis whether the development of a chronic condition over the follow-up period (i.e., incident disease) was associated with changes in RMR differently from those participants who remained free from that condition. Each model included baseline age, sex, lean body mass, fat body mass, and longitudinal changes in the latter two variables as covariates. 

## 3. Results

### 3.1. Cross-Sectional Analyses 

**Figure 1:**
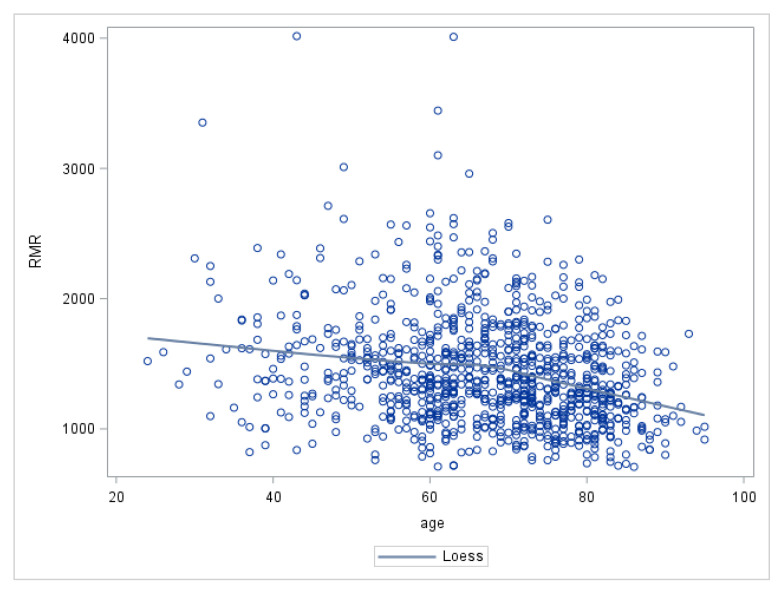
Scatterplot of the cross-sectional association between resting metabolic rate (RMR) (kCal/day) and age (years).

Table 1 reports the main characteristics of the study population: The mean age was 65.9 years, men and women were represented almost equally, 84.2% of the subjects had college education, and the most frequent ethnicity was Caucasian (67.6%). The mean RMR at baseline was 1607 kCal/day for men and 1269 kCal/day for women. As shown in Figure 1, RMR was progressively lower with increasing age.

**Figure 2:**
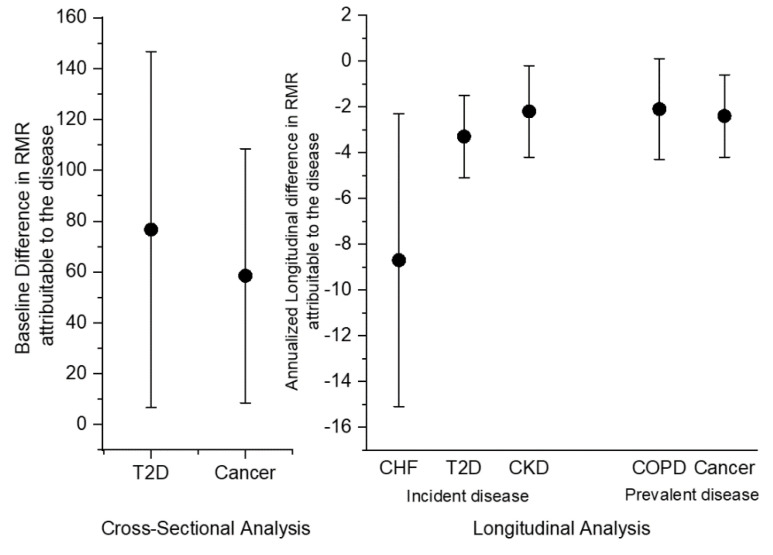
Differences in RMR attributable to diseases (Beta Coefficients ± 95% Confidence Intervals), as resulting from the cross-sectional and statistical analyses. Incident disease: absent at baseline and developed during follow-up; Prevalent disease: present at baseline. T2D, type 2 diabetes; CHF, chronic heart failure; CKD, chronic kidney disease; COPD, chronic obstructive pulmonary disease.

Results of the regression model assessing associations of RMR with 15 selected chronic conditions, after accounting for age, sex, and body composition, are shown in Table 2. T2D and cancer were positively and significantly correlated with RMR values, as represented in Figure 2. Noteworthy, older age and both lean and fat mass were significantly associated with RMR (*p* < 0.001), although on average the value of the β coefficients per kg of estimated lean body mass was 4-fold higher than the coefficient for fat mass, a finding that is consistent with other data reported in the literature [26,27,28].

### 3.2. Longitudinal Analyses

**Figure 3:**
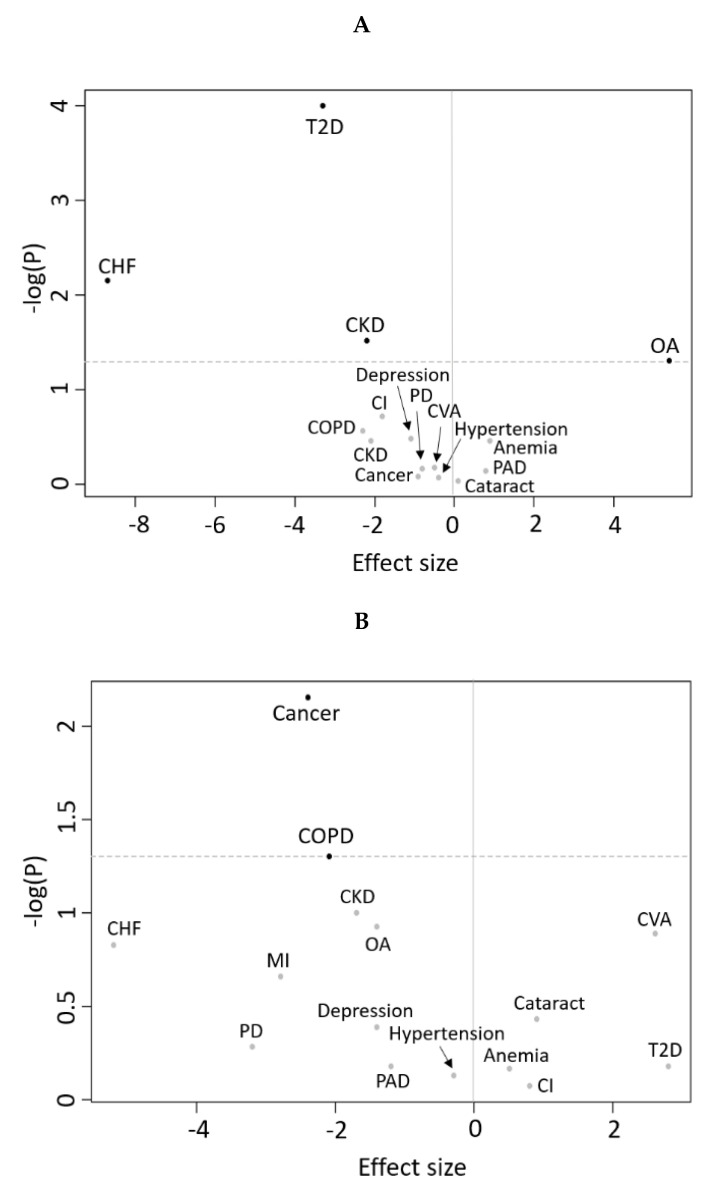
Volcano plots of the association between diseases and RMR rate of change over time. In panel (**A**), subjects free of disease at baseline that developed the disease during the follow-up are considered in comparison to subjects that never developed the disease, adjusting for age, sex, lean and fat body mass, and changes in lean and fat body mass. In panel (**B**), subjects affected by the disease at baseline are considered in comparison to subjects that never developed that disease, adjusting for age, sex, lean and fat body mass, and changes in lean and fat body mass. The dotted line represents *p* value = 0.05. Effect size refers to the beta coefficient of the association between disease and RMR rate of change. CHF, chronic heart failure; MI, myocardial infarction; CVA, cerebrovascular accidents; T2D, type 2 diabetes mellitus; PAD, peripheral artery disease; CI, cognitive impairment; PD, Parkinson’s disease; CKD, chronic kidney disease; COPD, chronic obstructive pulmonary disease; OA, osteoarthritis.

The results of the longitudinal analyses are shown in Table 3 and Table 4, and in Figure 2 and Figure 3. Incident T2D, CHF, and CKD were associated with a steeper decline in RMR, independent of age, sex, and body composition (*p* < 0.05), while incident OA was associated with an increase in RMR with marginal significance (*p* = 0.05) (Figure 2 and Figure 3A).

Prevalent COPD and cancer were associated with a steeper decline in RMR, independent of age, sex, and body composition (Figure 2 and Figure 3B). No other incident or prevalent disease status was associated with significant differential change in RMR over time.

## 4. Discussion

### 4.1. Principal Findings 

In this study, we explored the cross-sectional and longitudinal associations of RMR and prevalent and incidence of major chronic diseases, independent of relevant covariates. Older age was associated with lower resting energetic expenditure at baseline, while baseline T2D and cancer were correlated with higher expenditure levels. Longitudinally, aging was associated with a decline in RMR over time. The presence of incident T2D, CHF, and CKD, as well as that of prevalent COPD and cancer, was also associated with a longitudinal decrease in RMR. Incident OA was associated with an increase in RMR.

### 4.2. Cross-Sectional Associations Between Disease Status and RMR

A number of studies have shown that chronic inflammation is associated with aging and age-associated chronic diseases and may contribute to increased catabolic rates and age-associated changes in body composition, including sarcopenia and muscle wasting [29]. Based on the results of this study, we hypothesize that muscle wasting, reduced physical activity, and inflammation are part of a vicious cycle that complicates the clinical course of many chronic diseases and could have different impacts on the energetic “cost of living” depending on the specific phase of the natural history of such diseases. In particular, while in the initial and overt state of clinical manifestations, conditions may stimulate a repair response that requires extra-energy to maintain or regain homeostatic equilibrium; in the long term, their profound impact on body composition, especially parenchymatous organs, which may not be fully captured by the available methods, may lead to substantial reduction of RMR.

The cross-sectional association between cancer and higher basal metabolism we observed is not surprising, since among the modifications that occur in a cancerous cell there is an increase in energy needs to support accelerated growth and survival [30]. Cancerous tissue competes for nutrients with neighboring tissues and this is believed to be the main cause of the progressive and often deleterious cachexia that commonly occurs in patients with malignant tumors. Furthermore, a very tight connection exists between cancer development and progression and inflammation, which could constitute another mechanism of elevated energy metabolism [31].

The findings regarding the cross-sectional association between T2D and increased basal metabolism are consistent with previous studies [32,33]. Several mechanisms have been proposed to explain this association, including increased protein turnover, futile substrate cycling, gluconeogenesis, plasma glucagon concentrations, and sympathetic nervous system activity [34]. Inflammation has been tightly connected to the development of insulin resistance and T2D, therefore constitutes another potential explanation for augmented energetic expenditure [35,36].

Another hypothesis that could explain the increased basal metabolism observed in these conditions is the accumulation of senescent cells. Cell senescence is the defense mechanism triggered by genomic instability due to the exposure to a stressor, such as oxidative and genotoxic insults, chromatin perturbation, oncogene activation, mitochondrial dysfunction, or protein misfolding [37]. The cell stops replicating, withdrawing from the cell cycle, and develops specific features such as resistance to apoptosis, increased energy metabolism, and production of bioactive molecules globally defined as “Senescence Associated Secretory Phenotype” (SASP) [38]. SASP includes several pro-inflammatory proteins that determine damage to tissues and produce a chronic inflammatory environment [39]. Cell senescence and SASP have been involved in the pathogenesis of several age-related conditions, such as atherosclerosis [40], COPD [41], OA [42], Alzheimer’s disease [43], sarcopenia [44], and others [45]. We argue that the enhanced metabolic activity we observe in this analysis for some diseases could be attributable to the presence of increasing numbers of metabolically active senescent cells.

### 4.3. Disease Status and Longitudinal Changes in RMR

Expectedly, CHF was associated with a steeper decline in RMR as CHF has long been connected with a deleterious wasting syndrome, termed cardiac cachexia, a strong prognostic factor for CHF [46]. The pathogenesis of this wasting is unclear, as it has been linked mostly to neurohormonal and immunological changes, but also to malnutrition and malabsorption [47]. Nevertheless, it is clear that CHF is characterized by a complex anabolism/catabolism imbalance towards the latter [48]. Loss of body mass with CHF, and of muscle mass in particular, could be the process underlying the decrease in RMR over time observed in this analysis. Adjusting for body composition in our statistical models did not change this association. However, DEXA may capture macroscopic volumetric differences in the various compartments but not more subtle structural modifications that may profoundly impact metabolism and energy consumption. The same concept could be applied to other diseases showing an association with declining RMR including CKD, cancer and COPD, which are among the main causes of cachexia and muscle dysfunction [49,50]. Although changes in body composition are part of the natural history of these diseases, the measured changes did not account for the decline in RMR, which could be related to a more intrinsic tissue or cellular alteration. Another possibility is that in the case of cancer, the adjustment for lean mass determined a statistical “overadjustment” due to the progression of cancer and growth of abnormal non-fatty tissue. T2D is also tightly associated with modifications in body composition and has been related to impaired muscle strength and quality [51].

Conceivably, diseases characterized by intense fatigue, shortness of breath (heart failure, COPD, cancers affecting the respiratory system), peripheral neuropathy, and infections (T2D) can lead to exercise intolerance. Reduced physical activity determines a loss of the most metabolically active muscle fibers, muscle denervation and atrophy, muscle infiltration of connective tissue, and a decrease in mitochondrial oxidative capacity [52,53]. Notably, our group recently showed that mitochondrial oxidative capacity of the skeletal muscle is directly associated with RMR, independent of muscle mass or density [54]. 

OA was the only condition associated with an increase in RMR over time, with marginal significance (*p* = 0.05). OA is characterized by an inflammatory pathogenesis and has been correlated with an accumulation of senescent cells, both of which are potential mechanisms leading to the increased energy expenditure we observed, as discussed previously [42,55].

### 4.4. Limitations and Conclusions 

A possible interpretation of our findings is that the combined effect of chronic diseases and aging exerts a two-phase differential effect on body composition and energy consumption. A progressive impairment of metabolic homeostasis caused by chronic diseases that have systemic effects, such as CHF, COPD and diabetes, challenges energy production; and the availability of energy at the cellular, parenchymal, and organismal level is a fundamental requirement for resiliency strategies. This progressive imbalance between damage accumulation and its functional consequences and shrinking of reserve capacity is non-linear. In the initial response phase, the RMR increases because strategies to maintain a homeostatic equilibrium are energetically more expensive than normal physiological mechanisms of a healthy person. With depletion of reserve capacity, the extra-energy is no longer required, energy is scarce, and catabolism becomes the prevalent process, with complex changes in body composition not fully captured by traditional technology. This theory is consistent with many studies showing a rapid decline in body weight and blood pressure in the years prior to death [56,57]. Figure 1. helps visualize metabolic decline with aging and how that could be accelerated by the constant demands of chronic diseases.

To the best of our knowledge, no previous research has examined the longitudinal relationship between the incidence of 15 chronic diseases typical of the aging population and trends in basal energy expenditure over time. Although the mechanisms that link energy metabolism to the pathogenesis and the natural history of specific diseases appear to be complex and far from understood, this study provides an inkling of the directionality and strength of this relationship and allows some early speculation regarding underlying pathways.

The limitations of this study concern the exceptionally healthy and well-educated status of the BLSA participants relative to the general population, and the unstable interval between visits. Moreover, complete information regarding all the parameters considered were unavailable for all the subjects: depending on the testing needed for a specific diagnosis, the population considered for each disease varied slightly. Finally, some pathologies were significantly less prevalent than others, leading to a lower statistical power in evaluating their association with the outcome of interest. However, we consider our study to provide solid and novel information in the complex and broad field of energy metabolism in aging.

The effect of changes in body composition during disease progression appeared to affect RMR with a magnitude that could not be accounted for by adjustments for DEXA measurements. DEXA is unable to discriminate the contractile, metabolically active components of muscle tissue from other molecules, mainly of connective tissue, that tend to accumulate with aging. Utilization of more precise techniques, such as dilution of deuterated creatine; whole-body potassium counter; and high-resolution, three-dimensional computed topography, should be considered in future studies to reliably assess the contribution of skeletal muscle to RMR [58]. 

Further research on larger populations is needed to confirm these results and obtain more complete information. Outlining the pathways through which a pathological condition affects the energetic balance of the human body could provide more complete knowledge about the condition itself, an increased awareness of the metabolic trends in healthy aging in contrast to health deterioration, and a better understanding of the mechanisms employed to contrast or respond to an insult. Furthermore, defining a specific pattern of progressive metabolic change resulting from an active pathological process could eventually allow clinical inferences in relation to staging and prognosis. If the metabolic alteration proves to be a sign of the body fighting against the disease, and the features of this connection can be fully characterized, the ability to directly measure this battle through RMR may provide an excellent tool to assess the response of a specific individual to a particular stressor.

## Figures and Tables

**Table 1:** Characteristics of the population at baseline (average ± standard deviation).

Number of Participants	997 (489 Males + 508 Females)
Age (years)	65.9 ± 12.9
Race, % White	67.6
% Black	26.2
% Other	6.2
Education, % Completed College	84.2
Current Smoking, %	2.0
Resting Metabolic Rate (kCal/day)	1607 ± 429.6 in males, 1268.9 ± 331.9 in females
Physical Activity (min/week)	93.9 ± 137.7
Height (cm)	168.9 ± 9.3
Body Mass Index (kg/m^2^)	27.0 ± 4.6
Fat Body Mass (kg)	27.0 ± 10.4
Lean Body Mass (kg)	47.5 ± 10.0

**Table 2:** Multivariable Linear Regression models testing the relationship of prevalent disease with resting metabolic rate at baseline, adjusting for age, sex, and body composition. Sex was coded as 1 for male, 0 for female.

Disease	Prevalence at Baseline (%)	β Value	*p* Value
		Estimate (SD)	
Chronic Heart Failure	1	175.1 (110.8)	0.11
Sex		26.2 (49.6)	0.6
Age		−4.9 (1.0)	<0.001
Lean Mass		22.2 (2.5)	<0.001
Fat Mass		5.2 (1.3)	<0.001
Myocardial Infarction	2.6	55.9 (68.1)	0.41
Sex		47.4 (44.3)	0.28
Age		−5.11 (0.9)	<0.001
Lean Mass		20.2 (2.2)	<0.001
Fat Mass		5.9 (1.1)	<0.001
Cerebrovascular Accident	5.1	−32.1 (50.7)	0.52
Sex		44.7 (46.3)	0.335
Age		−5.4 (1.0)	<0.001
Lean Mass		20.6 (2.3)	<0.001
Fat Mass		6.0 (1.2)	<0.001
Hypertension	40.2	23.3 (2.9)	0.9
Sex		48.2 (44.5)	0.28
Age		−5.1 (0.9)	<0.001
Lean Mass		20.2 (2.2)	<0.001
Fat Mass		5.9 (1.2)	<0.001
Type 2 Diabetes Mellitus	10.7	76.7 (36.0)	0.04
Sex		32.7 (44.7)	0.46
Age		−5.4 (0.9)	<0.001
Lean Mass		20.7 (2.2)	<0.001
Fat Mass		5.5 (1.2)	<0.001
Anemia	12.1	−12.8 (33.6)	0.7
Sex		49.2 (44.3)	0.27
Age		−5.0 (0.9)	<0.001
Lean Mass		20.1 (2.2)	<0.001
Fat Mass		6.0 (1.2)	<0.001
Peripheral Artery Disease	1.8	−31.9 (82.6)	0.7
Sex		46.8 (44.9)	0.3
Age		−5.0 (0.9)	<0.001
Lean Mass		20.3 (2.3)	<0.001
Fat Mass		5.9 (1.2)	<0.001
Cognitive Impairment	0.6	−47.2 (138.2)	0.73
Sex		48.3 (46.4)	0.3
Age		−5.3 (1.0)	<0.001
Lean Mass		20.5 (2.3)	<0.001
Fat Mass		6.0 (1.2)	<0.001
Depression	4.4	26.2 (51.6)	0.61
Sex		48.5 (44.3)	0.27
Age		−5.0 (0.9)	<0.001
Lean Mass		20.2 (2.2)	<0.001
Fat Mass		6.0 (1.2)	<0.001
Parkinson’s Disease	0.5	23.9 (150.6)	0.87
Sex		48.2 (44.4)	0.28
Age		−5.1 (0.9)	<0.001
Lean Mass		20.2 (2.2)	<0.001
Fat Mass		6.0 (1.2)	<0.001
Chronic Kidney Disease	24.4	31.8 (28.6)	0.71
Sex		58.2 (45.1)	0.562
Age		−5.4 (1.0)	<0.001
Lean Mass		20.2 (2.2)	<0.001
Fat mass		6.0 (1.1)	<0.001
Cataract	22.9	−9.5 (27.6)	0.73
Sex		49.5 (44.3)	0.26
Age		−5.0 (1.0)	<0.001
Lean mass		20.1 (2.2)	<0.001
Fat mass		6.0 (1.2)	<0.001
Chronic obstructive pulmonary disease	13.9	44.9 (31.4)	0.15
Sex			
Age		50.7 (44.3)	0.25
Lean mass		−5.1 (0.9)	<0.001
Fat mass		20.0 (2.2)	<0.001
		5.9 (1.2)	<0.001
Cancer	25.9	58.5 (25.7)	0.02
Sex		44.4 (44.2)	0.32
Age		−5.6 (0.9)	<0.001
Lean mass		20.2 (2.2)	<0.001
Fat mass		6.1 (1.2)	<0.001
Osteoarthritis	22.1	36.7 (26.6)	0.17
Sex		52.4 (44.3)	0.24
Age		−5.3 (0.9)	<0.001
Lean mass		20.0 (2.2)	<0.001
Fat mass		5.8 (1.2)	<0.001

**Table 3:** Linear mixed effect models comparing rate of change over time of RMR (Kcal/day) in subjects with new onset of disease with subjects that never developed the disease. The models are adjusted for baseline age, sex, baseline lean body mass, fat mass, and their rate of change over time.

Disease	β Value (SE)	*p* Value
Chronic Heart Failure	−8.7 (3.2)	0.007
Myocardial Infarction	−0.4 (2.1)	0.85
Cerebrovascular Accident	−0.8 (1.9)	0.68
Hypertension	−0.5 (1.1)	0.67
Diabetes Mellitus	−3.3 (0.9)	<0.001
Anemia	0.9 (1.0)	0.35
Peripheral Artery Disease	0.8 (2.1)	0.72
Cognitive Impairment	−2.1 (2.2)	0.35
Depression	−1.8 (1.4)	0.19
Parkinson’s Disease	−0.9 (4.0)	0.82
Chronic Kidney Disease	−2.2 (1.0)	0.03
Cataract	0.1 (0.9)	0.92
Chronic Obstructive Pulmonary Disease	−2.3 (2.1)	0.27
Cancer	−1.1 (1.1)	0.33
Osteoarthritis	5.4 (2.7)	0.05

**Table 4:** Linear mixed effect models analyzing rate of change over time in RMR (Kcal/day) in subjects with disease present at baseline as compared to RMR change in subjects that never developed the disease. The models are adjusted for baseline age, sex, baseline lean body mass, fat mass, and their rate of change over time.

Disease	β Value (SE)	*p* Value
Chronic Heart Failure	−5.2 (3.7)	0.15
Myocardial Infarction	−2.8 (2.3)	0.22
Cerebrovascular Accident	2.6 (1.7)	0.13
Hypertension	−0.3 (0.8)	0.75
Diabetes Mellitus	2.8 (6.5)	0.67
Anemia	0.5 (1.1)	0.69
Peripheral Artery Disease	−1.2 (2.8)	0.67
Cognitive Impairment	0.8 (4.6)	0.85
Depression	−1.4 (1.7)	0.41
Parkinson’s Disease	−3.2 (5.1)	0.52
Chronic Kidney Disease	−1.7 (1.1)	0.10
Cataract	0.9 (1.0)	0.37
Chronic Obstructive Pulmonary Disease	−2.1 (1.1)	0.05
Cancer	−2.4 (0.9)	0.007
Osteoarthritis	−1.4 (0.9)	0.12
